# Ossifying Fibromyxoid tumor of soft parts in head and neck: case report and literature review

**DOI:** 10.1186/s13000-018-0699-7

**Published:** 2018-03-27

**Authors:** Ignacio A. Velasco, Ran Zhang, Tiejun Li, Diancan Wang

**Affiliations:** 1 Department of Oral and Maxillofacial Surgery, Peking University School and Hospital of Stomatology, 22 South Zhongguancun Avenue, Beijing, 100081 People’s Republic of China; 2 Department of Oral Pathology, Peking University School and Hospital of Stomatology, Beijing, People’s Republic of China

**Keywords:** Ossifying Fibromyxoid tumor, Head and neck Neoplasms

## Abstract

**Background:**

Ossifying fibromyxoid tumor of soft parts (OFMT), is a rare but morphologically distinctive neoplasm of uncertain histogenesis that most frequently affects middle-aged male adults. Clinically, it usually presents as a slowly enlarging, small, circumscribed mass, which in most cases is painless. OFMT is most frequently found within the subcutaneous tissues of extremities or trunk, and rarely in the oral/head and neck region. We present an unusual case of this tumor in the submandibular region, and, based on the current medical literatures this is probably the first case described in this anatomical location.

**Case presentation:**

A 32-year-old male presented to our outpatient clinic with a right submandibular mass with 1-year of evolution. Excisional biopsy showed that it was characterized by ossification along the periphery of the lesion. The neoplastic cells were spindle-like with scant eosinophilic cytoplasm. These cells were arranged with uniform cell-to-cell space in a fibromyxoid stroma. Small and large clusters of calcifications were present within the tumor. Immunohistochemically, the case showed positive staining of S-100 protein, vimentin, nestin, calponin, SMA, GFAF, desmin, INI-1, caldesmon, and CD34. It also showed negative staining of CK, CK7, CK8/18, NF, and EMA. About 2% of neoplastic cells showed positive staining of Ki67. Based on these features, the final pathological diagnosis was OFMT.

**Conclusions:**

It is hoped that a greater understanding of OFMT in the head and neck region will avoid potential misdiagnosis, and contribute to determining the correct management, which appears to be complete surgical excision with close follow-up for recurrence surveillance.

## Background

Ossifying fibromyxoid tumor of soft parts (OFMT) was first described by Enzinger et al. in 1989, and is a rare but morphologically distinctive neoplasm of undetermined histogenesis [1]. OFMT most frequently affects middle-aged adults, with a slight male predominance [2]. Clinically, it usually presents as a slowly enlarging, small, circumscribed mass, which in most cases is painless, although it can infrequently cause symptoms such as pain and paresthesia [3]. OFMT is most frequently found within the subcutaneous tissues of the extremities or trunk, and is rarely found in the oral/head and neck region [2–6].

OFMT is typically composed microscopically of lobules of small polygonal to spindle-shaped cells with vesicular nuclei, discernible nucleoli, and eosinophilic cytoplasm, arranged in cords, trabeculae, or clusters in a loose fibromyxoid matrix [3]. Although most OFMTs fit this histologic description and show consistently benign clinical behavior, a subgroup of OFMT displays atypical histopathologic features, such as high cellularity or increased mitotic activity, and show a more aggressive clinical behavior [3]. In the vast majority of cases, complete surgical excision is curative; however, recurrences and distant metastasis have been reported in cases that presented with high mitotic activity and cytologic atypia [2, 3, 5].

The objective of our study is to describe an unusual clinical presentation of this tumor in the submandibular region. Based on the current medical literatures, this is probably the first case described in this anatomical location. Additionally, a literature review was performed with particular emphasis on previously reported cases affecting the oral/head and neck region.

## Case presentation

**Fig. 1:**
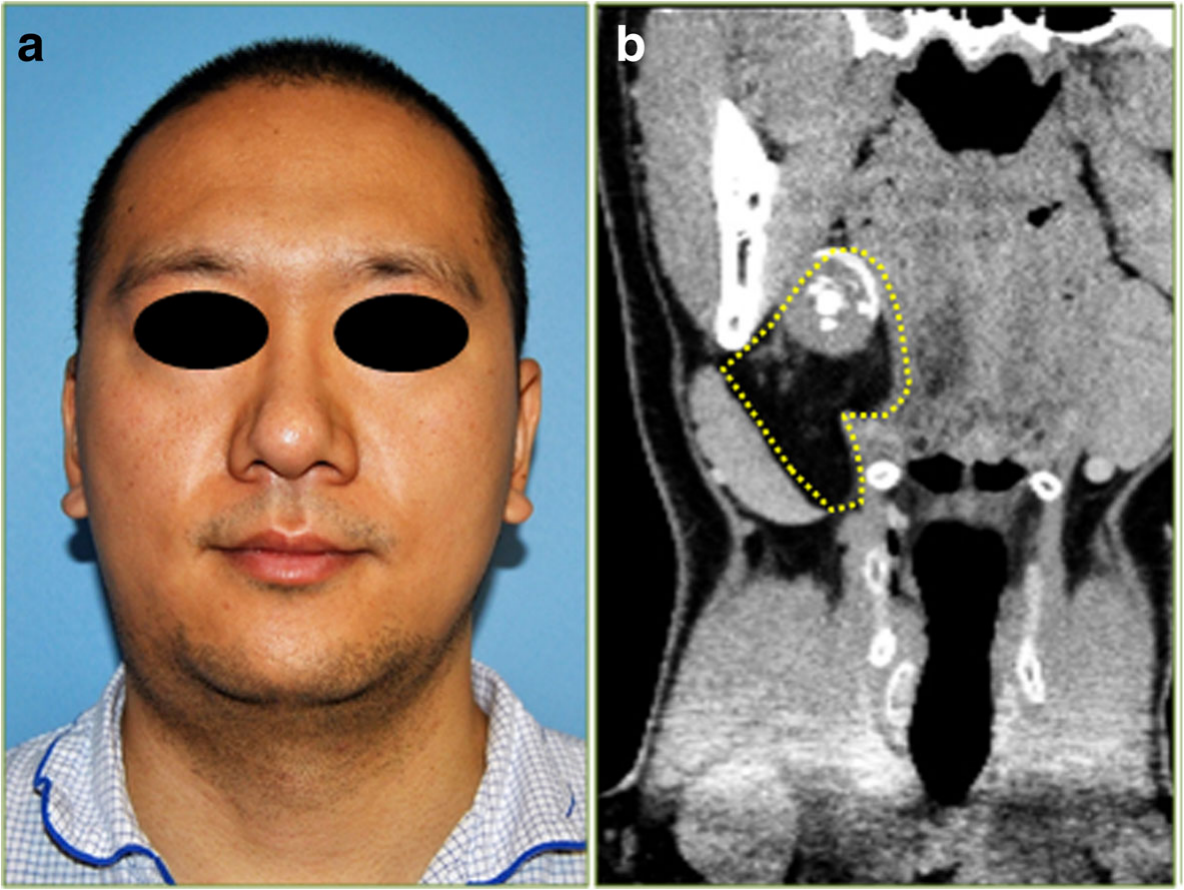
Extraoral photograph shows a mass in the patient’s right submandibular region (**a**). Coronal head CT scan shows a mixed lesion (outlined by the dotted line) near the right submandibular gland (**b**)

**Fig. 2:**
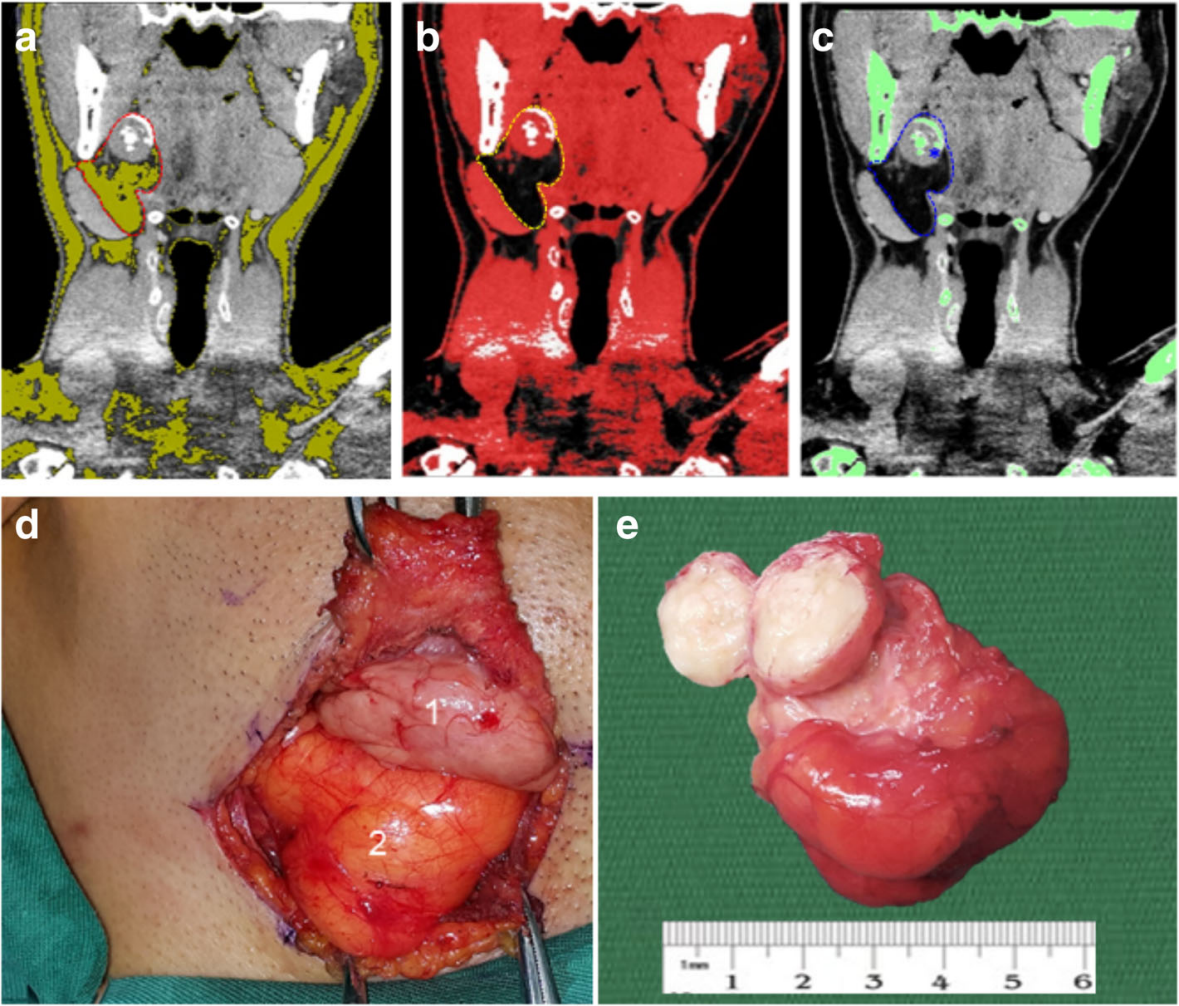
Additional tissue masks were made using the image viewer revealing that the tumor was possibly composed of fat (yellow) (**a**), connective tissues (red) (**b**), and osteoid materials (green) (**c**) as outlined by the dotted line. Intraoperative photograph shows submandibular gland (1), lesion (2) (**d**). Gross specimen of the lesion after the surgery (**e**).

A 32-year-old Asian male patient presented to the outpatient clinic of Peking University Hospital of Stomatology (Beijing, China) with1-year history of a right neck mass; the patient’s past medical history was non-contributory. Extraoral clinical examination revealed a soft and slightly movable mass in the right submandibular region with normal overlying skin (Fig. 1a); the remaining physical examination results were within normal limits. The patient underwent computed tomography (CT), and the data were processed using the image viewer InVesalius 3.0.0 version (Centre for Information Technology Renato Archer, Campinas, SP, Brazil). CT imaging showed a large (5 × 3 cm^2^) mixed lesion with defined margins that was adjunct to the right submandibular gland (Fig. 1b). Additional tissue masks were created using the image viewer and these revealed that the tumor was possibly composed of fat, connective, and osteoid tissues (Fig. 2a–c). The primary differential diagnoses considered for this lesion included pleomorphic adenoma of the submandibular gland, teratoma, and osteo/chondroid lipoma.

Excisional biopsy was performed through an extraoral approach with submandibular gland preservation (Fig. 2d). Gross specimen dissection revealed the presence of hard tissues in the mass (Fig. 2e) and intraoperative frozen sections tested negative for malignancy. The postoperative period was uneventful and there were no signs of recurrence in the first 6 months of follow-up.

## Material and methods

The tissue sections were fixed in formalin, embedded in paraffin (the thickness is 5 μm) and then were stained with haematoxylin–eosin and immunohistochemistry. Following heat-induced epitope retrieval, slides were incubated with antibodies specific for: S-100, nestin, calponin, vimentin, cytokeratin(CK), cytokeratin 7 (CK7), CK8/18, calponin, smooth muscle actin (SMA), glialfibrillary acidic protein (GFAP), neurofilament (NF), desmin, caldesmon, CD34, epithelial membrane antigen (EMA) and INI-1 (Dako, Denmark; Zhongshanjinqiao, China). Detection of the immunoreactionwas performed using second antibodies and ZLI-9017/9018/9019 DAB kit (both from Zhongshanjinqiao, China).

The literature review was performed using the PubMed/MEDLINE electronic database to identify relevant publications from January 1989 through to December 2016 using the following search terms: “ossifying fibromyxoid tumor AND head and neck neoplasms.” From the publications obtained in this search, those pertaining to cases of OFMT in the oral/head and neck region were included in the review [1, 4–26]. Additionally, a manual search was conducted by cross-referencing the retrieved manuscripts. All available data were reviewed, including clinical presentation, histopathologic examination findings (cytologic atypia/mitotic activity), and surgical intervention (Table 1).

**Table 1:** Summary of OFMT cases in the oral/head and neck region reported from 1989 to 2016

Reference	Patients (n)	Age (y)	Sex	Location	Clinical presentation	Surgical treatment	Mitotic activity	Atypia	Recurrence	Metastasis	Follow-up
Enzinger FM et al. (1989) [1]	8	14–79 (mean 47)	64% M	Head and neck, various sites	Slow growing painless mass	Local excision (majority of cases)	N/D	N/D	N/D	No	1–32 y (mean 6.2)
Schofield JB et al. (1993) [7]	4	41	M	Inner cheek	Slow growing mass	N/D	–	–	No	No	1–10 y (median 7)
		78	M	Neck	Slow growing mass	N/D	N/D	N/D	No	No	
		39	M	Lip	Slow growing mass	N/D	0–11/10 HPFs	–	No	No	
		49	M	Pretracheal	Slow growing mass	N/D	N/D	N/D	No	No	
Williams SB et al. (1993) [4]	9	51	F	Posterior neck	Slow growing mass	Local excision	–	–	No	No	2 y
		52	M	Submental area	Slow growing mass	Local excision	–	–	No	No	N/D
		39	M	Chin	Slow growing mass	Local excision	–	–	No	No	2 y
		67	F	L mandible vestibule	Slow growing mass	Local excision	–	–	No	No	1.5 y
		29	M	L lateral max and nasal bone	Slow growing mass	Local excision	–	–	No	No	1.5 y
		37	M	Soft palate	Slow growing mass	Local excision	–	–	No	No	3 y
		66	M	Scalp	Slow growing mass	Local excision	–	–	No	No	2 y
		75	M	L neck	Slow growing mass	Local excision	–	–	No	No	1 y
		58	F	L parapharynx	Slow growing mass	Local excision	+	+	Yes (2)	No	2.1 y
Williams RW et al. (1994) [8]	1	35	M	Parotid/zygomatic arch region	Slow growing mass	Wide local excision	+	+	Yes (3)	No	24 y
Ng WK et al. (1995) [9]	1	52	M	R nostril, middle meatus	Swelling, intermittent epiphora	N/D	N/D	N/D	N/D	N/D	N/D
Thompson J et al. (1995) [10]	1	35	M	L nasal cavity	Congestion and pain	Partial removal	–	–	Yes	No	N/D
Lax S et al. (1995) [11]	1	50	M	R thyroid lobe	Nodular enlargement	Total thyroidectomy	+	–	No	No	3 y
Zamecnik M et al. (1997) [12]	2	71	M	Neck	Subcutaneous mass	Surgical removal	+	+	Yes (2)	No	4 y
		45	F	Neck	Subcutaneous mass	Surgical removal	+	+	Yes	Yes (lung)	DOD
Ekfors TO et al. (1998) [13]	2	63	M	Neck	Subcutaneous mass	N/D	+	–	No	No	N/D
		2	F	Head	Subcutaneous mass	N/D	–	–	No	No	N/D
Paschen C et al. (2001) [14]	1	12	M	Nasal cavity and paranasal sinus	Nasal congestion	Local excision	–	–	No	No	N/D
Folpe AL et al. (2003) [15]	9	14–80 (median 49)	56% M	Head and neck, various sites	Subcutaneous mass	Wide excision (majority of cases)	1 patient	–	Yes (2 in single patient)	1 patient (leg)	5–240 mo (mean 57)
Al-Mazrou KA (2004) [16]	1	3-wk. infant	M	L nasomaxillary fold	Slight fullness	Local excision	–	–	No	No	6 mo
Mollaoglu N et al. (2006) [17]	1	13	M	L side of mandible	Rapid swelling	Local excision	–	–	No	No	p
Park DJ et al. (2006) [18]	1	81	F	R orbit	Diplopia, pain, swelling	Local excision	–	–	Yes (2)	No	6 y
Seykora JT et al. (2006) [19]	1	67	F	Scalp	Multilobular and cystic mass	Wide local excision	2/10 HPFs	–	No	No	8 y
Suehiro K et al. (2006) [20]	1	38	F	Scalp	Subcutaneous mass	Local excision	5/10 HPFs	–	Yes (3)	Yes (lung, brain)	DOD
Blum A et al. (2006) [21]	1	49	F	Nasal septum	Nasal congestion and swelling	Local excision	–	–	No	No	1 mo
Miliaras D et al. (2007) [22]	1	39	M	Mandibular symphysis skin	Slow growing, subcutaneous mass	Local excision	–	–	No	No	1 y
Hirose T et al. (2007) [23]	2	42	M	Nasal vestibule	N/D	Local excision	+	+	Yes (2)	No	17 y
		54	M	L supraclavicular region	Small nodule	Local excision	–	–	No	No	6 mo
Sharif MA et al. (2008) [24]	1	14	F	Between buccal and gingival mucosa	Slowly growing gingival mass	Local excision	–	–	No	No	N/D
Miettinen M et al. (2008) [25]	20	21–81 (median 50)	62% M	9 neck, 3 scalp, 1 lower lip, 7 various sites	Variable	Local excision	0–41/50 HPFs	–	22% (≥1)	No	2–61 y (mean 13)
Nonaka CF et al. (2009) [6]	1	21	F	R posterior mandibular gingiva	Painless exophytic mass	Local excision	–	–	No	No	7 mo
Graham RP et al. (2011) [33]	9	39–63 (median 52)	52% M	Head and neck, various sites	N/D	N/D	N/D	N/D	N/D	N/D	12–149 mo
Kondylidou-Sidira et al.(2011) [5]	1	24	M	Zygomatomaxillary buttress	Subcutaneous lump	Local excision	–	–	No	No	2 y
Gebre-Mehdin et al. (2012) [28]	4	47	F	Temple	N/D	N/D	–	–	No	No	N/D
		59	M	Neck	N/D	N/D	N/D	+	Yes (1)	Yes (rib)	N/D
		42	M	Neck	N/D	N/D	N/D	+	No	No	N/D
		43	M	Paralaryngeal	N/D	N/D	N/D	+	No	No	N/D
Ohta et al. (2013) [31]	1	26	M	Tongue	Painless mass	Local excision	> 2/10 HPFs	–	Yes (1)	No	4 y
Ottoman B (2015) [30]	1	72	M	L posterior maxilla	Exophytic mass	N/D	–	–	No	No	N/D
Dantey K et al. (2016) [26]	1	33	M	Parotid region	Mass	Local excision	–	–	No	No	3 y
Case report	1	52	M	R submandibular region	Right neck swelling	Local excision	–	–	No	No	6 mo

*Abbreviations*: *M* male, *F* female, *R* right, *L* left, *N/D* not described, *y* years, *mo* months, *wk.* week, *DOD* died of disease

## Results

### Case presentation

**Fig. 3:**
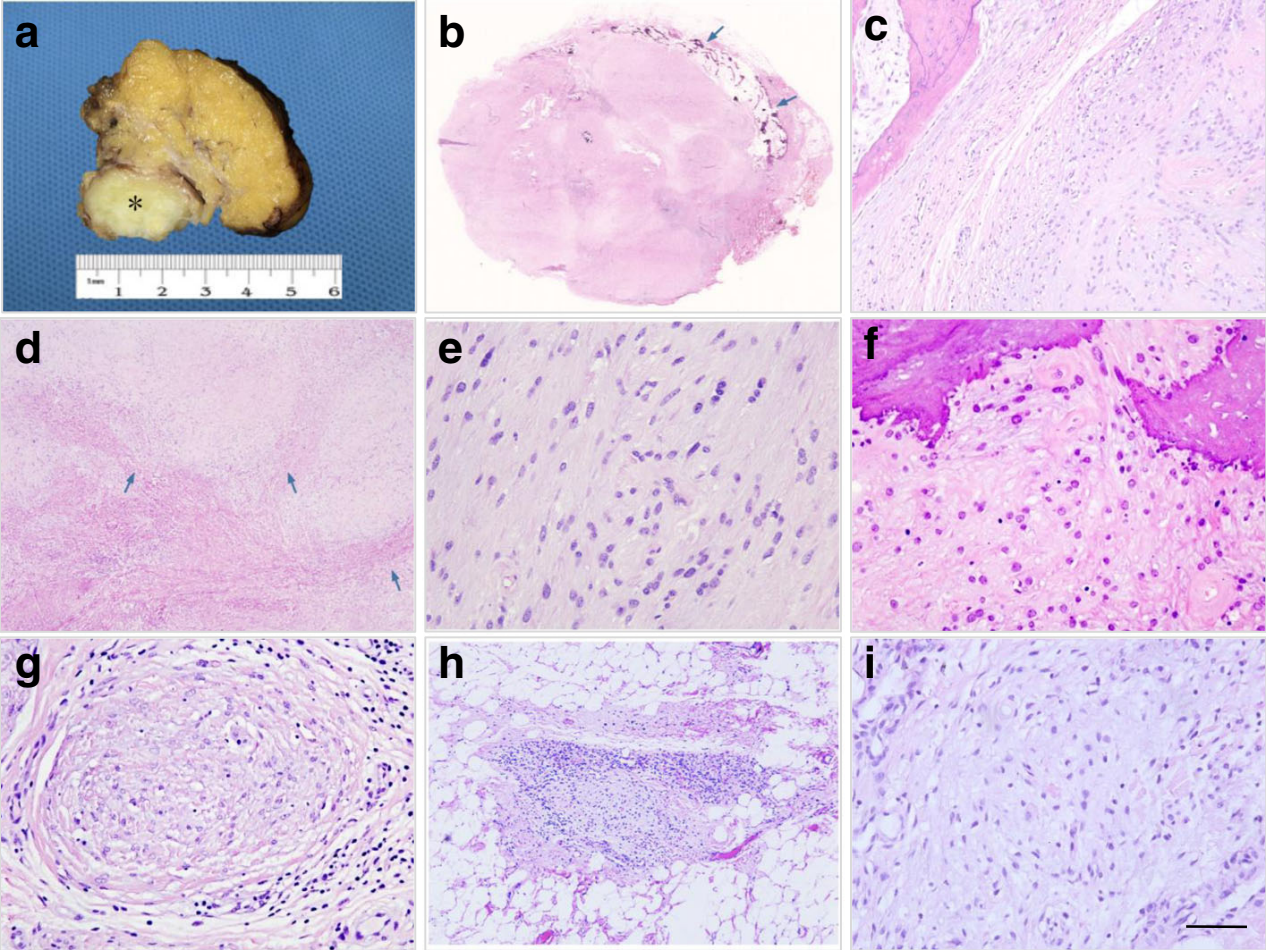
Ossifying fibromyxoid tumor. The cut surface of the tumor is oval, gray-white and well-demarcated from the adjacent soft tissues with delicate fibrous septa (asterisk indicates) (**a**). The tumor is associated with a peripheral shell of metaplastic bone (arrows indicate) (**b**, **c**). Fibrous septa can be seen from the capsule into the neoplasm, separating them into cellular islands (**d**). Cells in this case are typically uniform, they are polygonal or spindle shape with fibromyxoid-appearing matrix (**e**). Clusters of calcification are within the tumor (**f**). Nodules of tumor cells can be seen outside of the capsule (**g**). Sections of the soft tissues adjacent to the tumor show clusters of tumor cells (**h**, **j**). Scale bar: 250 μm (**d**), 100 μm (**h**),50 μm (**c**), 25 μm (e, f, g, i)

**Fig. 4:**
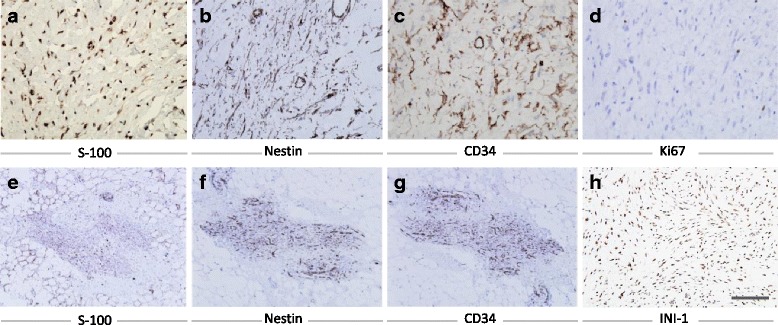
Images of immunohistochemical results of ossifying fibromyxoid tumors. Lesion cells stain positive for S-100, nestin, CD34 and INI-1 (**a-c, h**). 2% lesion cells stain positive for Ki67 (**d**). Small lesions in the tissues adjacent to the tumor show positive stain for S-100, nestin and CD34 (**e-g**). Scale bar: 100 μm (**e-g**), 50 μm (**h**), 25 μm (**a-d**)

Examination of the biopsy specimen showed a 5.5 cm × 5 cm × 3 cm, rubbery, fragile, gray-white colored, well-defined tumor that was surrounded by dark yellow lobulated soft tissues (Fig. 3a). Histologically, ossification was present along the periphery of the lesion (Fig. 3b–c). The neoplastic cells were separated by fibrous septa and arranged in nests and cords (Fig. 3d). Neoplastic cells were spindle-like with scant eosinophilic cytoplasm. These cells were arranged with uniform cell-to-cell space in a fibromyxoid stroma (Fig. 3e). Small and large clusters of calcifications were present within the tumor (Fig. 3f). The neoplasm was closely associated with the glands, which were composed of dominant mucous components and suspected to be sublingual glands or minor salivary glands. Outside of the neoplasm, nodules of neoplastic cells had invaded into the adjacent tissues (Fig. 3g). Small clusters of lesion cells were found in the adjacent soft tissues (Fig. 3h and i). Immunohistochemically, the case showed positive staining of S-100 protein, vimentin, nestin, INI-1, calponin, SMA, GFAP, desmin, caldesmon, and CD34. It also showed negative staining of CK, CK7, CK8/18, NF, and EMA (Fig. 4a–c, h). About 2% of neoplastic cells showed positive staining of Ki67 (Fig. 4d). Small lesions in the adjacent soft tissues showed similar immunohistochemical staining patterns in immunohistochemistry (Fig. 4e–g). Based on these features, the final pathological diagnosis was OFMT.

### Literature review

Twenty-eight articles with well-documented OFMT cases affecting the oral/head and neck region were reviewed; all articles were published between 1989 and December 2016. Table 1 describes the epidemiology, clinical presentation, surgical treatment, and outcome of this sample population, in addition to one new case that was reported (*n* = 88). According to the data reviewed, OFMT in the head and neck region usually presents as a painless subcutaneous mass in middle-aged men. OFMT in the oral cavity was seen in 8 of 88 cases (9%) and a less common location was the nasal cavity (4/88, 5%), where it was associated with pain and nasal congestion. The preferred surgical treatment in the majority of cases was local excision; however, more radical excisions were performed in recurrent disease. Increased mitotic activity of > 2 per 10 high-power fields (HPFs) and cytological atypia was seen in 12/45 (27%) and 8/68 (12%) cases, respectively. At least 1 episode of recurrence was described in 15 of 70 cases (21%) and was associated in almost all cases with an increased mitotic activity of > 2 per 10 HPFs (14/15, 93%). Distant metastasis was a rare event that occurred in only 4 of 78 cases (5%), and in all cases, it was associated with recurrent disease and increased mitotic activity. Two patients with distant metastasis died of the disease.

## Discussion

OFMT is a rare musculoskeletal tumor of borderline malignant potential [2, 27]. The etiopathogenesis and exact line of differentiation of OFMT is presently unclear [27]. Evidence exists for schwannian or neuronal differentiation, but this has not been well proven [12, 27–29]. Similarly, cartilaginous or myoepithelial differentiation was proposed by Enzinger et al. [1] and Kilpatrick et al. [30]. From these hypotheses, Graham et al. [27] suggested a “scrambled” phenotype for these tumors.

Since the first description by Enzinger et al. [1], several case reports and series have documented OFMT in various anatomical locations. Men are affected more frequently than women, with a wide age range of 1–81 years (median, 50 years) [1, 4–8, 10–26]. The head and neck region is an infrequent anatomical location for OFMT; in our literature review, we identified 87 cases, and we report 1 further case in a unique location within the submandibular gland, which was the first OFMT case in our institution. Oral lesions are even rarer and have been reported to occur in the gingiva, palatal mucosa, and tongue. A total of 8 confirmed cases of oral cavity OFMT have been published [4–7, 17, 24, 25, 31].

The clinical presentation of OFMT in the head and neck does not differ much from that in other anatomical regions. Head and neck OFMT presents as a well-circumscribed, slow-growing, painless, and subcutaneous/submucosal mass. However, nasal cavity OFMT usually presents as pain and nasal congestion. These tumors normally have a longstanding clinical course, ranging from 1 to 20 years, or even longer (median, 4 years) [3, 32]. On radiographic examination, OFMT presents as a nodular soft tissue mass with an incomplete peripheral rim of ossification [27, 33]. CT reveals a peripheral “bone shell” in at least 60% to 70% of cases [15, 27]. In our reported case, the lesion presented with a mixed phenotype with peripheral hard tissue composition.

OFMT histopathology is characterized by the presence of uniform lobules, round to fusiform-shaped cells arranged in nests and cords, and set in a variably fibromyxoid stroma [27]. Approximately 70% of lesions are surrounded by an incomplete shell of metaplastic (hypocellular) lamellar bone, and the other 30% lack the bone shell (non-ossifying variant) [27, 33]. Our reported case was surrounded by a shell of lamellar bone. OFMT are typically positive for S-100 and vimentin (70%), often show desmin positivity, and can also express Leu-7, neuron-specific enolase, glial fibrillary acidic protein, and α-SMA [27, 33]. From the immunohistochemical and ultrastructural findings, a preponderance of evidence has suggested that OFMT is of neuroectodermal origin [12, 27, 29, 30]. Vimentin and S-100 staining is often positive and can be recommended as a useful adjunct to determine the pathologic diagnosis. Genetic analyses have demonstrated that INI-1 gene (22q) is deleted in a small number cases of the OFMT, and immunohistochemiscal stainning for INI-1 was lost completely or in a mosaic pattern, however, in our case we didn’t detect this staining pattern [34]. Nonetheless, an accurate histopathologic diagnosis of OFMT is essential to distinguish this tumor from its differential diagnoses, which include epithelioid nerve sheath tumors, such as epithelioid schwannoma, mixed tumors/myoepitheliomas, extraskeletal myxoid chondrosarcomas, and osteosarcomas [2, 27]. None of the cases of epithelioid schwannomas have the bone shell and cell-cell space is not uniform, as is seen in OFMT. Furthermore, epithelioid schwannomas often arise near a nerve, while this is rare in OFMT cases [35]. Mixed tumors/myoepitheliomas do not usually have surrounding bone; however, they have evident epithelial differentiation and show positive staining of epithelial markers, such as cytokeratins, which are not expressed in OFMT [36]. Extraskeletal myxoid chondrosarcomas are extremely rare in the head and neck region. They contain eosinophilic cells that are arranged in nests and cords with hemorrhage in some areas [37]. Osteosarcomas seldom grow in a lobular pattern, and have much more cytologic atypia and pleomorphism than the malignant form of OFMT [38].

Atypical and malignant OFMT have been described based on certain histopathologic criteria [2, 15, 27]. However, there are different opinions regarding the metastatic potential of the malignant subtype [1, 27]. Furthermore, three microscopic subtypes of OFMT have been previously described, namely, typical, atypical, and malignant, based on cellularity, nuclear grade, and mitotic activity. Folpe et al. [15] described three subtypes of OFMT (typical, atypical, and malignant); tumors that presented with a high grade or high cellularity and a mitotic rate of ≥ 2 mitoses per 50 HPFs were categorized as malignant OFMT, as such cases were found to be associated with distant metastasis. Other groups of tumors that did not present with the features of typical OFMT, but also did not meet the parameters for malignancy, were classified as atypical subtypes [15, 27]. In contrast, Miettinen et al. [2] reported tumors without increased mitotic activity that metastasized.

Recurrences have been reported in a considerable number of OFMT cases [1]. The clinical and histologic features of the recurrent tumors have not been consistently different from those of nonrecurring lesions [5]. An increase in cellularity and greater mitosis has been observed in some of the recurrent tumors, which is supported by our review. The incidence of recurrence of head and neck-specific OFMT has been previously described by Kondylidou-Sidira et al. [5] and we obtained the same value of 21%. In our case, mitotic activity, cytologic atypia, and pleomorphism were rarely seen under the microscope; however, extracapsular growth of the tumor cells was obvious, indicating the multi-focal growth pattern. These surrounding tumor cells shared similarities in morphology and staining pattern with the cells within the tumor itself. Fat tissues were observed around the tumor which was a signature in this case and it had aroused some speculations that whether the tumor was originated from the fat tissues or not. To answer this question we need constant accumulation and observation of cases in the future. Besides, further investigation is required to determine whether this type of growth pattern is associated with a risk of recurrence. Therefore, predictors of the malignancy and recurrence of OFMT are subjects of research and discussion in the future.

Surgical management is the initial treatment of choice for head and neck OFMT. Local excision is described as the primary therapy, which is curative in most cases. However, close follow-up is recommended, especially in atypical and malignant histopathologic types, because of the previously discussed propensity for local recurrences and distant metastases. In recurrent cases, wide and more radical excisions with secure margins would be the appropriate treatment. Adjuvant radiotherapy does not seem to be indicated, except for the management of distant metastasis or unresectable disease.

## Conclusions

It is hoped that a greater understanding of OFMT in the head and neck region will avoid potential misdiagnosis, and contribute to determining the correct management, which appears to be complete surgical excision with close follow-up for recurrencesurveillance.

## Abbreviations

CK: Cytokeratin
CT: Computed tomography
DOD: Died of disease
EMA: Epithelial membrane antigen
F: Female
GFAP: Glial fibrillary acidic protein
HPF: High-power field
L: Left
M: Male
mo: Months
N/D: Not described
NF: Neurofilament
OFMT: Ossifying fibromyxoid tumor of soft parts
R: Right
SMA: Smooth muscle actin
wk.: Week
y: Years
